# Resident Immunity in Tissue Repair and Maintenance: The Zebrafish Model Coming of Age

**DOI:** 10.3389/fcell.2019.00012

**Published:** 2019-02-05

**Authors:** Raquel Rua Martins, Pam S. Ellis, Ryan B. MacDonald, Rebecca J. Richardson, Catarina Martins Henriques

**Affiliations:** ^1^Department of Oncology and Metabolism, Medical School, University of Sheffield, Sheffield, United Kingdom; ^2^Bateson Centre, University of Sheffield, Sheffield, United Kingdom; ^3^Department of Infection, Immunity and Cardiovascular Disease, University of Sheffield, Sheffield, United Kingdom; ^4^School of Physiology, Pharmacology and Neuroscience, University of Bristol, Bristol, United Kingdom

**Keywords:** adult zebrafish (Danio Rerio), tissue resident immunity, tissue repair and regeneration, ageing, gut, heart, retina, brain

## Abstract

The zebrafish has emerged as an exciting vertebrate model to study different aspects of immune system development, particularly due to its transparent embryonic development, the availability of multiple fluorescent reporter lines, efficient genetic tools and live imaging capabilities. However, the study of immunity in zebrafish has largely been limited to early larval stages due to an incomplete knowledge of the full repertoire of immune cells and their specific markers, in particular, a lack of cell surface antibodies to detect and isolate such cells in living tissues. Here we focus on tissue resident or associated immunity beyond development, in the adult zebrafish. It is our view that, with our increasing knowledge and the development of improved tools and protocols, the adult zebrafish will be increasingly appreciated for offering valuable insights into the role of immunity in tissue repair and maintenance, in both health and disease throughout the lifecourse.

## Introduction

It is becoming increasingly clear that the innate and adaptive immune systems play crucial roles in tissue maintenance and repair during health and disease. Studies in animal models are crucial to identifying complex functions of immunity in sometimes surprising aspects of biology. For example, it was discovered relatively recently that macrophages, previously thought of as purely cell debris-eating machines, promote fibrosis and scarring in mammals after an injury. Further, they have been identified as being crucial for tissue regeneration, directly communicating with epithelial cells in a variety of vertebrate models, reviewed elsewhere (Pott and Hornef, 2012; Ginhoux and Guilliams, 2016). Zebrafish are well placed as a model to decipher the complex functions of immune cells in tissue regeneration and other disease related processes due to their genetic tractability and the ease of live imaging. However, the majority of studies are largely limited to embryonic and larval stages due to their rapid, external development, genetic tractability, and transparent embryonic development. However, to best study tissue regeneration and human disease, fully differentiated tissues and organs are required. Here we put forward the adult zebrafish as a relevant and valid model for studying tissue immunity in health and disease throughout the whole animal’s lifecourse. We highlight the recent advances in our knowledge of tissue immunity in adult zebrafish and the best tools currently available to study it. It is our view that our increasing knowledge and the on-going development of tools and protocols are already making the adult zebrafish a valuable model offering insights into the role of immunity in tissue health throughout the lifecourse, and this model is likely to become more and more eminent in the future of the field, if we push forward for the continuous development of tools.

### Ontogeny of Tissue Immunity in Zebrafish

It wouldn’t make sense to delve into adult zebrafish tissue immunity before addressing their ontogeny. Unfortunately, though, this is where the problem starts. In the mouse, the most commonly used vertebrate immunology model, the origin of tissue resident or associated immune cells is generally well described, exemplified in (Bain et al., 2014; Ginhoux and Guilliams, 2016; Ferrero et al., 2018), whereas in the zebrafish, our knowledge is still largely incomplete.

In mice, extensive work over decades has shown that most tissues have resident immune cells, both innate (mainly macrophages and NK cells, depending on the tissue) and adaptive (T- and B-cells). The different flavors within these immune cells vary depending on tissue and disease status (Mowat et al., 2017; White et al., 2017). Amongst these, we know the most about macrophages. In mice, tissue-resident macrophages seem to derive from embryonic precursors that populate most tissues during embryogenesis, becoming a specialized, tissue-resident, self-renewing population in the adult (Hoeffel et al., 2012; Hashimoto et al., 2013; Yona et al., 2013; Hoeffel and Ginhoux, 2015). A well-known exception, at least in mice, is the gut. Recent work has shown that the macrophage population in the adult mouse gut is constantly re-populated by circulating monocytes, which then differentiate into mature macrophages and are maintained *in situ* (Bain et al., 2014; Bain and Mowat, 2014a,b). In zebrafish, our knowledge is more limited. Nevertheless, recent work by Alemany et al. has identified distinct signatures in resident immune cells in the adult zebrafish, using sophisticated single-cell sequencing and tracking analysis (Alemany et al., 2018). Their work shows that haematopoietic cells in the kidney marrow derive from a small set of multipotent embryonic progenitors. Surprisingly, the authors indicate that resident immune cells in the fin do not originate from haematopoietic stem cells and instead seem to arise either from epidermal and mesenchymal transdifferentiation, or from ectodermal ancestors similarly to mesenchymal cells. The origin and maintenance of resident immune cells remains to be fully elucidated in other organs such as the gut. Notwithstanding, the zebrafish model is also making key contributions to the understanding of tissue immunity in vertebrates, thanks to an impressive availability of transgenic reporter lines for different immune cells/inflammatory markers (see Table 1 for details). Seminal work has shown that, like in other vertebrates, zebrafish have a fully functional tissue-associated immunity, including T-cells, B-cells, macrophages, neutrophils, eosinophils, and mast cells (Moss et al., 2009; Renshaw and Trede, 2012; Nguyen-Chi et al., 2015; Pereiro et al., 2015; Dee et al., 2016), even if it is not yet determined whether they are resident in all tissues or not. Emerging data, however, is shedding light on the ontogeny of tissue immunity in zebrafish.

**Table 1:** Selected list of key transgenic zebrafish lines and antibodies to detect immune cells and/or inflammation markers.

Promoter	Immune cell type	Commercial source	Reference	Technical notes: conditions that work	Conditions that don’t work
*pu.1*	Primitive myeloid cells		Hsu et al., 2004		
*mpx*	Neutrophils		Renshaw et al., 2006; Mathias et al., 2006		
*lyz*	Neutrophils/some macrophages		Hall et al., 2007		
*gata2a*	Eosinophils		Traver et al., 2003		
*mpeg1*	Macrophages		Ellett et al., 2011		
*c-fms*	Macrophages		Dee et al., 2016		
*mfap4*	Macrophages		Walton et al., 2015		
*tnfα*	Pro-inflammatory, *tnfα*+ cells		Marjoram et al., 2015; Nguyen-Chi et al., 2015		
*lck*	T-cells		Langenau et al., 2004		
*cd4*	CD4^+^ T-cells and macrophages		Dee et al., 2016		
*foxp3a*	Foxp3a+ T-cells		Hui et al., 2017		
*rag2*	Lymphoid cells		Langenau et al., 2003.		
*mhc2dab, cd45*	Allows distinction between B-cells, macrophages/dendritics and T-cells/neutrophils		Wittamer et al., 2011		
*IgM*	B-cells		Page et al., 2013.		
*CD79*	B-cells		Liu et al., 2017		
*apoe*	Microglia		Oosterhof et al., 2017		
**Antibodies**					
Rabbit anti L-plastin	Pan-leukocyte	Ref: GTX124420, Genetex	Redd et al., 2006.	1:300/1:500 on whole mount tissue, paraffin and cryosections with Citrate pH6 antigen retrival	cryosections without citrate pH6 Ag retrieval
Rabbit anti-Mpeg1 (C terminus)	Macrophages	Ref: ANA55917, AnaSpec Inc		1:50 on cyosections and (weakly) on paraffin sections, both after *in situ* hybridisation heating steps. Note that, at least in our hands, in adult zebrafish gut, this antibody does not dettect all of mpeg-mcherry transgenically labeled macrophages	
Rabbit anti-TCR-alpha (N terminus)	T-cells	Ref: AS-55868, AnaSpec Inc	Our unpublished data (see Figure 1)	1:200 on cryosections(In our hands, we noticed a significamnt decline in the quality of staining iof the cryosections were not fresh, i.e, frozen for more than a week or so) with citrate pH6 Ag retrieval	
Isolectin GS-IB4	vascular endothelial cells and microglia	Thermo Fisher I21411	Zou et al., 2013	Cryosections or paraffin sections witjh Citrate Ph6 antigen retrieval	
Mouse anti-7.4C4	microglia (vascular-derived, resident macrophages)		Becker and Becker, 2001	Cryosections or paraffin sections witjh Citrate Ph6 antigen retrieval (1:100 dilution)	
Mouse anti-RFP		Ref: ABP-MAB-RT008, Insight biotechnology		1:500 on paraffin or cryosections with citrate pH6 Ag retrieval	
Chicken anti-GFP		Ref: AB13970, Abcam		1:500 on paraffin or cryosections with citrate pH6 Ag retrieval	
Mouse anti-Glutamine Synthetase	Muller Glia	MAB302 clone GS6	Gramage et al., 2015	Cryosections with citrate Ph6 antigen retrieval 1:200 dilution	
Rabbit Spi1 spleen focus forming virus (SFFV) (Pu.1)	progenitor myeloid cells	GTX128266-S Genetex			In our hands (CMH) we did not achieve clear results in either paraffin or cryosections with or without antigen retrieval
Goat anti-EPX (eosinophil specific peroxidase)	Eosinophils	SC-19148 Santa Cruz Biotechnology			In our hands (CMH) we did not achieve clear results in either paraffin or cryosections with or without antigen retrieval
Purified Rat Anti-Mouse CD11b Clone M1/70 (RUO)	Multiple leukocyes	557394 BD			In our hands (CMH) we did not achieve clear results in either paraffin or cryosections with or without antigen retrieval

Recent work has shown that microglia, the specialized macrophages in the Central Nervous System (CNS); have different origins depending on the age of the animals. In the adult zebrafish, microglia derive from haematopoietic stem cells (HSCs) and not from primitive macrophages, which occurs only in early development (Ferrero et al., 2018). This has also been shown for adult zebrafish Langerhans cells in the skin and suggested to also be the case for liver, heart, gut and brain (He et al., 2018). Together, these elegant recent studies suggest that most zebrafish adult resident or associated immunity derives from the second wave of hematopoiesis, mainly from the ventral wall of the dorsal aorta (VDA region), and not from erythro-myeloid progenitors (EMPs) as previously thought. This is also emerging as the current model in mammalian systems (Sheng et al., 2015) although there are still uncertainties and some controversy in the field (Perdiguero et al., 2015).

Crucially, recent work is showing that, more than ontogeny, tissue immunity seems to be particularly dictated by the tissue in which it resides. There are key tissues in adult zebrafish that are being intensely investigated and multiple studies highlight that the role of immunity in tissue repair and maintenance is largely conserved in zebrafish. Key examples where this has been shown are in the heart, gut, brain, and retina.

### Selected Examples of Tissue Immunity in Adult Zebrafish

#### Heart

**FIGURE 1:**
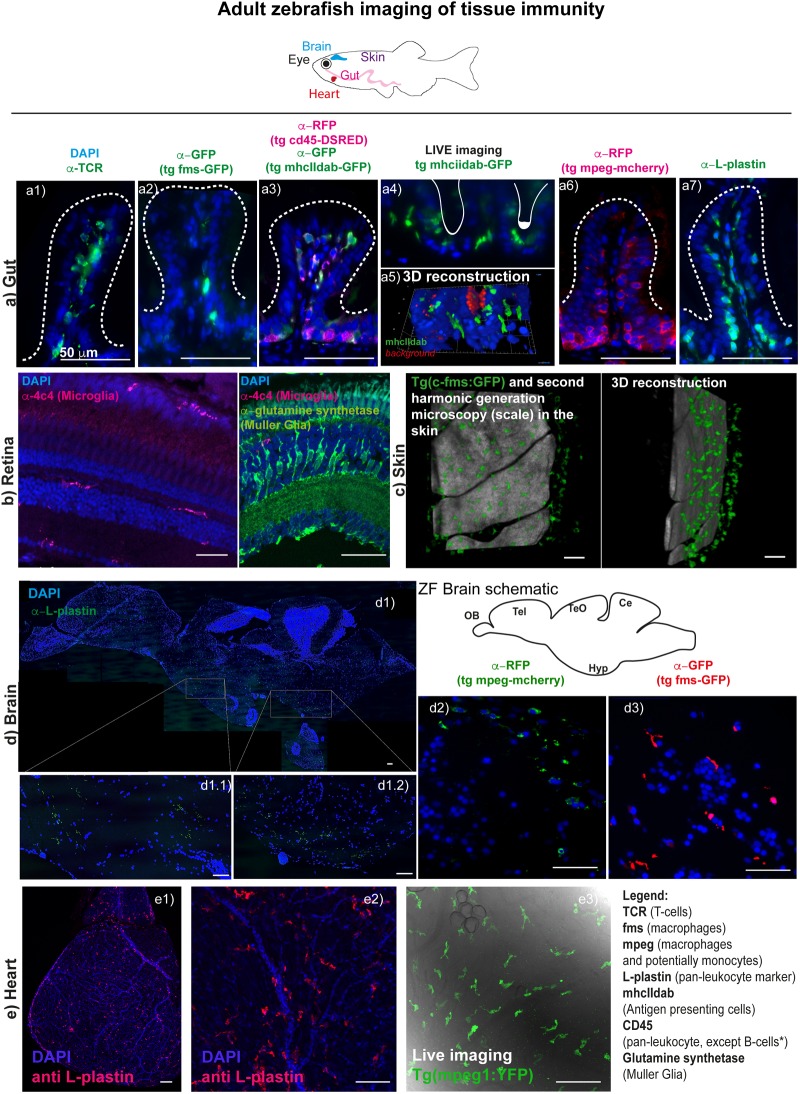
Imaging tissue immunity in adult Zebrafish. **(a)** CMH unpublished images showing examples of cryo/paraffin sections of adult Zebrafish gut, followed by immune fluorescence of selected antigens (*see table 1 for antibody details*), all counterstained with DAPI for cell nuclei detection and imaged in a Deltavision light microscope. **(a1)** T-cells are stained with anti-TCR alpha antibody **(a2)** macrophages in Tg(fms-GFP) animals (Dee et al., 2016) are detected with an anti-GFP antibody; **(a3)** multiple leukocyte lineages can be detected using anti-RFP and anti-GFP antibodies in a double transgenic line for CD45-DSRED and mhcIIdab-GFP respectively (Wittamer et al., 2011). Single red cells are neutrophils/T-cells, double green and red cells are macrophages/dendritic cells, single green cells are B-cells, since, in this line, the CD45 promoter used is not active in B-cells; **(a4)** and **(a5)** Adult Zebrafish gut can be imaged in Light sheet microscopy for short durations, following dissection and gentle embedding in low percentage agarose in E3 media; **(a6)** macrophages are detected with an anti-RFP antibody in Tg(*mpeg1: mCherryCAAX*) (Ellett et al., 2011; Ogryzko et al., 2018) animals; **(a7)** leukocytes are stained with L-plastin (aka LCP-1) and these can n = be seen lining the gut epithelia cells (enterocytes). **(b)** RBM unpublished images showing selected cryosections of adult Zebrafish retina, imaged by confocal microscopy followed by immune fluorescence of selected antigens (*see table 1 for antibody details*), all counterstained with DAPI for cell nuclei detection. **(b1)** Microglia are detected with an anti-4c4 antibody and can be seen dispersed throughout the tissue, displaying a simple ramified state. Upon insult these microglia rapidly migrate and engulf damaged cells or debris. **(b2)** Muller Glia can also be detected in the retina using an anti-glutamine synthetase antibody. **(c)** RJR unpublished images of adult zebrafish skin where macrophages can be live-imaged, shown here using the Tg(*c-fms-GFP)* line and using second harmonic generation microscopy to identify the scale surface. **(d)** RRM unpublished images showing paraffin sections of adult Zebrafish brain, imaged using a Deltavision light microscope or by confocal microscopy; **(d1)** multiple-panel reconstitution of a whole adult brain, imaged by confocal microscopy, stained with anti-L-plastin, which marks all microglia in the brain. Microglia in the adult brain can also be detected for example using the **(d1)** Tg(*mpeg1:mCherryCAAX*) line (Ellett et al., 2011; Ogryzko et al., 2018) or **(d2)** the Tg(fms-GFP) line (Dee et al., 2016). Sections **(d1)** and **(d2)** show the diencephalon. **(e)** RJR unpublished images showing whole mount immunostaining of an entire heart **(e1)** or the surface of the ventricle **(e2)** with an anti-L plastin antibody and imaged with confocal microscopy. Live imaging using confocal microscopy of an unwounded adult zebrafish heart reveals cardiac macrophages using a Tg(*mpeg1:YFP*) line **(e3)**.

Recent years have seen many studies identify crucial and perhaps surprising roles for immune cell populations in the heart in homeostasis and disease, although much remains to be discovered. A recent study in mice indicated a remarkable role for resident cardiac macrophages in the distal atrioventricular node where they make direct connections to cardiomyocytes via Connexin 43 and facilitate electrical conductance (Hulsmans et al., 2017). In zebrafish, we currently know very little about cardiac macrophages under homeostatic conditions although our own experience has revealed a population of immune cells, labeled with L-plastin and transgenic markers of macrophages (see Table 1), is present in the unwounded heart and recent work suggests these may be derived from HSCs (see above; Figure 1). Recently, many studies have shown important contributions of different immune cell lineages in response to cardiac injury and disease in mammalian models. In particular, vital roles have been suggested for macrophages in complete regeneration of the neonatal mouse heart (Aurora and Olson, 2014; Lavine et al., 2014). However, the inflammatory response in the adult zebrafish heart has been less well characterized. Recent studies revealed that immune cells are recruited to the heart following cryoinjury of the ventricle in adult zebrafish (Schnabel et al., 2011). Two recent reports have also shown that macrophages are required for cardiomyocyte proliferation and therefore regeneration in the heart of adult zebrafish (de Preux Charles et al., 2016; Lai et al., 2017). Our own experience suggests that all immune cell lineages that we were able to analyse are recruited to the heart after injury and whereas roles can be assigned for macrophages of the innate immune system, the precise roles for other cell types remain more of a mystery (RJR, unpublished). However, another recent report has shown important roles for a Treg subset of zebrafish T-cells in promoting regeneration in a number of different tissues including the heart (Hui et al., 2017), suggesting intriguing and important roles for different immune cell populations in varying aspects of regeneration and disease remain to be elucidated.

#### Gut

The gut can be considered the biggest compartment of the immune system, and it is constantly exposed to multiple foreign antigens, which it must distinguish from harmless dietary proteins and the resident microbiota (Mowat, 2018). When this goes wrong, the immune system can “misfire” and contribute to chronic inflammatory disorders such as Inflammatory Bowel Disease (IBD) (Bain and Mowat, 2014b; Mowat et al., 2017; Andrews et al., 2018; Corridoni et al., 2018; Liu et al., 2018) and age-associated gut degeneration (Man et al., 2014; Sato et al., 2015; Soenen et al., 2016). Additionally, gut immunity is essential for the steady-state epithelial renewal (Andrews et al., 2018) that, similarly to humans, occurs roughly every 3 days in adult zebrafish (Wallace et al., 2005; Crosnier et al., 2006). Recovery after DSS intestinal injury has also been shown to be dependent on Myd88 signaling in myeloid cells (Malvin et al., 2012). Adult zebrafish are also showing great promise as a model to study gut inflammation and repair in health and disease (Marjoram and Bagnat, 2015 Brugman, 2016), including ageing (Henriques et al., 2013; Carneiro et al., 2016, Martins et al., 2018). These works have shown that, similarly to humans, critical aspects of gut homeostasis become compromised with ageing in zebrafish, namely increased permeability, inflammation and telomere-dependent cellular senescence.

Thanks to the development of key transgenic reporter lines and antibodies (Table 1), adult zebrafish gut has been shown to be populated by abundant T-cells, B cells, mast cells, macrophages, and dendritic cells in the normal steady-state context (CMH unpublished data and others) (Wittamer et al., 2011; Lewis et al., 2014), similarly to mammalian vertebrates (Figure 1). Importantly, macrophage M1 and M2-like subsets have also been identified in zebrafish, thanks to the development of mpeg-mcherry-TNFα-GFP double transgenic line. Using this line, M1 macrophages were characterized by high TNFα-GFP expression (mpeg+TNFα+), as well as expression of TNFβ, IL-1β, and IL-6 (Nguyen-Chi et al., 2015), which are well-known markers of M1-like macrophages in mammals. Moreover, these subsets were shown to respond to injury similarly to human macrophage subsets. Additionally, Il-1β reporter lines have recently been developed allowing visualization of cells expressing this pro-inflammatory cytokine (Hasegawa et al., 2017; Ogryzko et al., 2018). What is very different in the zebrafish gut, however, is the absence, as far as reported, of defined intestinal crypts and Peyer’s patches (Ng et al., 2005; Cheng et al., 2016). Despite the absence of Peyer’s patches there is a clear distribution of leukocytes along the adult zebrafish gut, lining the enterocytes, which could be considered analogous to the mucosal associated lymphoid tissue (MALT). The apparent absence of secondary lymphoid structures though, means that we still do not understand fully how antigen presentation occurs in zebrafish (Lewis et al., 2014). MHC class II-expressing, antigen presenting cells (APCs), namely macrophages, dendritics (DCs) and B-cells are, however, described and appear to function similarly to their mammalian counterparts (Lugo-Villarino et al., 2010; Wittamer et al., 2011, Lewis et al., 2014; Dee et al., 2016). See Table 1 for further details and references.

Nevertheless, and importantly, the protective immune status of the gut has also been shown to occur in zebrafish, and this has been largely attributed to the secretion of IL-10 by CD41+foxp3+ Treg-like T-cells in the gut, once again showing that these sophisticated immune-regulatory elements are already evolved in teleost fish (Dee et al., 2016; Hui et al., 2017). It has also been shown that in adult zebrafish, the gut is rapidly populated by eosinophils upon parasitic infections (Balla et al., 2010), highlighting another conserved response to infection in the gut.

#### Brain

The neuro-immunology field has been growing in recent years, highlighting the complex crosstalk between the immune system and the central nervous system (CNS) and how this plays a key role in maintaining brain homeostasis, reviewed in (Oosterhof et al., 2015). Although mammalian models are more prominent at the moment, the zebrafish model is starting to gain traction, particularly due to its genetic and imaging amenity, small size and relatively low maintenance costs (de Abreu et al., 2018).

As in mammals (Cuadros and Navascues, 1998; Ginhoux et al., 2010), microglia are the tissue-resident immune cells in the zebrafish brain (Xu et al., 2015). In zebrafish, primitive microglia originate from yolk sac-derived macrophages that migrate to the cephalic mesenchyme and then invade the brain, being detectable from 60 h post-fertilization (hpf) (Herbomel et al., 2001). Only more recently it was recognized that these are not the definitive microglia observed in adulthood. As described above, an elegant recent study (Ferrero et al., 2018), showed the existence of a second wave of re-population of the brain’s microglia, which originate from HSCs – a process that occurs between 2 weeks and 3 months of age. Microglia can be detected by L-plastin (or LCP-1), a pan-leukocyte marker (Redd et al., 2006; Cvejic et al., 2008; Mathias et al., 2010; Van Houcke et al., 2017), by macrophage-expressed gene 1 (*mpeg1*), labeling all mononuclear phagocytes (Ellett et al., 2011; Wittamer et al., 2011), by the 4c4 antibody (Becker and Becker, 2001)as well as Apolipoprotein E (ApoE) [Oosterhof et al. (2015), see Table 1; Figure 1]. Note that, although ApoE specifically labels microglia if compared with the other markers mentioned, it has also been shown to be expressed astrocytes (Boyles et al., 1985; Poirier et al., 1991; Xu et al., 2006), oligodendrocytes (Stoll et al., 1989), as well as by some neurons albeit at lower levels (Han et al., 1994; Achariyar et al., 2016). Additionally, cells in the choroid plexus as well as smooth muscle cells in blood vessels also seem to express ApoE (Xu et al., 2006). Thus, in order to ensure specific labeling of microglia, ApoE should be used in combination with another leukocyte or macrophage marker.

Microglia have been described as ramified cells that constantly sense the environment searching for physiological disturbances in the surroundings (Oosterhof et al., 2015). Like in mammals (Lucin and Wyss-Coray, 2009), adult zebrafish microglia proliferate and migrate to the injury or inflammation site (‘gliosis’), upon activation in response to a stab lesion (Kroehne et al., 2011; Kyritsis et al., 2012), excitotoxin injection (Skaggs et al., 2014) or nitroreductase (NTR)-mediated neuronal ablation (Oosterhof et al., 2017). Also, there is an increased number of L-plastin^+^ cells in response to optic nerve injury in both young (5 months) and older (22–24 months) zebrafish, but this is decelerated in the old fish, suggesting age-related dysfunctional immune response in ageing (Van Houcke et al., 2017). Once activated, microglia change their appearance from a ramified to an amoeboid shape (Svahn et al., 2013). These immune cells have the important function of clearing cellular debris, such as dead or damaged neurons, by phagocytosis (Peri and Nusslein-Volhard, 2008); and, when activated can release anti- and pro- inflammatory cytokines, at least in mice primary microglia cultures (Cai et al., 2017). To our knowledge, microglia inflammatory cytokine release remains to described for zebrafish, despite extensive characterization of other aspects of zebrafish microglia (van Ham et al., 2014). Peripheral immune cells can infiltrate the CNS in cases of Blood Brain Barrier (BBB) alterations, such as those observed in Multiple Sclerosis (MS) or cerebral ischemia (Holtmaat and Caroni, 2016), in particular, infiltration of monocytes or perivascular macrophages has been described in mammals (Lucin and Wyss-Coray, 2009). Similarly, upon NTR-induced cell death, peripheral macrophage-like cells infiltrate the embryonic zebrafish brain, contributing to the first inflammatory response (van Ham et al., 2014). In opposition, it has been reported that no major infiltration of periphery macrophages occurs in the brain after neuronal ablation (Oosterhof et al., 2017). Thus, more studies are needed to address this question. More surprisingly, T cells were reported to infiltrate the brain in a mouse model of ALS (SOD1^G93A^) during progression of the disease (Chiu et al., 2008) and to invade the human brain in Parkinson’s Disease (PD) (Brochard et al., 2009). Also, CD4^+^ T cells and B cells have been detected in the brain of patients with MS, and this is thought to contribute to inflammation in the CNS (Jelcic et al., 2018). To our knowledge, so far, there are no studies reporting the presence of T cells or B cells in zebrafish brain. Zebrafish Treg-like (zTreg) cells seem to move towards damaged sites in CNS, such as retina and spinal cord, contributing to regeneration; however, the brain was not explored in this study (Hui et al., 2017). Moreover, it remains unknown whether neutrophils invade the adult zebrafish brain in contexts of severe inflammation. Neutrophils were found in the brain of a nlrc3-like mutant model zebrafish embryo, where there is a systemic inflammation (Shiau et al., 2013). However, no recruitment of neutrophils was observed after injury either in the embryo brain (van Ham et al., 2014) or peripheral nervous system (Pope and Voigt, 2014). Additionally, Goldshmit et al. reported to rarely find neutrophils at the injury site after spinal cord transection in adult zebrafish (Goldshmit et al., 2012). Unfortunately, though, other studies have yet to be reported for adult zebrafish to help clarify this matter.

#### Retina

The retina is viewed as a unique “window” into the brain and is one of the most established systems to study neural development and disease processes in the CNS (London et al., 2013). The zebrafish retina is a true vertebrate retina as it has the same organisation and contains largely the same types of neurons and glial cells as the human eye. The innate immune system in the zebrafish retina is composed of two major types of glial cell, the Müller glia (MG) and the microglia (Figure 1). The mammalian retina also houses astrocytes that will contribute to immunity. However, their presence in the zebrafish CNS, including the retina, remains unclear (Lyons and Talbot, 2014). The MG and microglia will contribute to the maintenance of homeostasis, phagocytose debris and are critical for tissue repair (Reichenbach and Bringmann, 2013). MG are the most abundant glial cell in the tissue, have a fixed radial morphology which allows them to contact surrounding neurons (Jadhav et al., 2009) and can modulate innate retinal immunity (Kumar et al., 2013; Vecino et al., 2016). Retina microglia are migratory, as in the brain, and survey the tissue for damage and debris (Silverman and Wong, 2018). Crosstalk between these two glial cell types may mediate their response to damage and injury by coordinating inflammation (Wang et al., 2011, 2014). Activated MG and microglia are associated with almost every pathological condition in the retina (Bringmann et al., 2006; Silverman and Wong, 2018). This includes retinal degenerative conditions, such as age related macular degeneration and diabetic retinopathies (Ramirez et al., 2017). The zebrafish is an established model for studying cellular and molecular mechanisms underlying many ocular diseases (Gestri et al., 2012). However, linking immunity with confounding factors for disease, such as ageing, remain challenging in many models. A recent study in zebrafish has shown that there is progressive degeneration of photoreceptors with age when interfering with Crumbs, a gene family linked with human retinal degeneration (Fu et al., 2018). However, the contribution of the innate immune system to degeneration and pathologies of disease remains largely unknown.

After damage the innate immune systems plays a key role in the phagocytosis of debris and removal of dead or dying cells (Kumar et al., 2013). However, in the zebrafish retina after damage or disease the MG will generate neurons to restore vision (Hitchcock and Raymond, 2004). This is an area of intense study and the molecular mechanisms regulating it are beginning to be identified (Goldman, 2014), yet the role of microglia in these processes is not clear (Mitchell et al., 2018). By imaging the glial dynamics in real time *in vivo* in the zebrafish retina, microglia have been shown to change their morphology to the activated state and maintain this activation after regeneration is complete, potentially to ensure correct retinal function is re-established (Mitchell et al., 2018). Further, by pairing the imaging capacity of the zebrafish with the ease to which they can be treated with pharmacological inhibitors a recent study investigated roles of the innate immune system during rod photoreceptor regeneration (White et al., 2017). They show that the role of microglia is to regulate MG responsiveness to cell death, and thereby control neuronal regeneration kinetics. Further, immunosuppression can either inhibit or accelerate photoreceptor regeneration kinetics depending on the timing of treatment (White et al., 2017). Thus, utilizing the precise advantages of the zebrafish, paired with the well-characterized retina, makes this an exciting model to study the resident immune system in retinal disease and regeneration.

## Concluding Remarks

Despite multiple advances in developing reporter transgenic lines marking different types of immune cell lineages in zebrafish, there are still multiple sub-types of immune cells we have no markers for or antibodies available e.g., mast cells. Nevertheless, advances in single-cell sequencing technology have already enabled the identification of specific immune subsets, such as different subtypes of NK cells (Carmona et al., 2017; Tang et al., 2017) and innate lymphoid cells (ILCs) (Hernandez et al., 2018), which have contributed to the understanding of the similarities and differences between zebrafish and human immune subsets. Despite the overall similarity between human and zebrafish immune subsets, highlighted here, there are key differences, which are important to keep in mind, reviewed elsewhere (Trede et al., 2004; Renshaw and Trede, 2012, Kanwal et al., 2014). The first obvious difference is that during the first week of zebrafish development, this organism relies entirely on an innate immune system (Lam et al., 2004), a difference which has been extensively used to understand the relative contribution of innate versus adaptive immunity in response to different bacterial, viral, and fungal pathogens (Meijer and Spaink, 2011; Meijer et al., 2014). Another key difference is the absence, at least not reported thus far, of secondary lymphoid organs in zebrafish. Moreover, the zebrafish does not have a bone marrow, and instead, T-, B- as well as myeloid cells are present in the spleen and head kidney, which act as the zebrafish equivalent of bone marrow. There are also key differences in zebrafish immune receptors and/or response to specific ligands reviewed in (Kanwal et al., 2014) and this is contributed to by the gene duplication detected in many of the zebrafish genes (Lu et al., 2012) An example are the novel immune-type receptors (NITRs), which appear to be homologues of mammalian NK-like receptors and seem to also have homologous functions (Yoder et al., 2010). Additionally, despite the fact that most of Toll Like receptors have been described in zebrafish, there are key differences such as the fact that Tlr4 is not involved in sensing LPS. Indeed, in zebrafish, LPS signals via a Tlr4- and MyD88-independent manner (Sepulcre et al., 2009). Nevertheless, zebrafish still respond to lipopolysaccharide (LPS), and careful analysis has shown that the overall response to LPS stimulation at the level of gene transcription is highly conserved with that of mammals (Forn-Cuni et al., 2017).

We have highlighted in Table 1 the working tools available as well as some antibodies that we have tested but have failed to get to work. We believe this will be a valuable starting point for future researchers wanting to use zebrafish to study tissue immunity.

In summary, we can clearly identify microglia, macrophages (including distinguishing a pro-inflammatory phenotype), T-cells, B-cells, and neutrophils in tissues using a combination of transgenic lines and antibodies. It will be particularly important to develop these techniques further if we are to improve our live imaging capability, but also the ability to detect multiple immune lineages in the same tissue without requiring crossing multiple transgenic lines, which dramatically increases the time and cost of experiments. Unfortunately, we are still missing transgenic reporters and/or antibodies for some sub-types of T-cells (e.g., Th1, Th2, cytotoxic, and NKT), NK-cells and mast cells.

We hope that the studies highlighted here show how zebrafish can offer an incredible tool to study immunity and its role in tissue repair and maintenance, across the lifecourse, in a time and cost-efficient manner, and how it can improve so much more with the continuous investment, not only of this scientific community, which is growing, but also of commercial companies, particularly in the development and validation of zebrafish-specific antibodies.

## Ethics Statement

This study complied with the Animals (Scientific Procedures) Act 1986 using Home Office approved licenses [PPL numbers: 30/3318 (RJR), 70/8681 (CMH), and 40/3727 (RBM)]. The licenses and protocols were ethically reviewed and approved by each local Animal Welfare and Ethical Review Body (AWERB) (University of Sheffield and University of Bristol). Both Universities are signatories of the Understanding Animal Research Concordat on Openness and as Signatories to the Concordat have agreed to be more open about their use of animals in research, and to abide by the four commitments.

## Author Contributions

CMH, RRM, RBM, and RJR contributed equally to the writing of the manuscript, figure, and table. PSE contributed to the development of key techniques illustrated in Figure 1 and contributed to Table 1.

## Conflict of Interest Statement

The authors declare that the research was conducted in the absence of any commercial or financial relationships that could be construed as a potential conflict of interest.
